# *Andrias davidianus* Ranavirus (ADRV) Genome Replicate Efficiently by Engaging Cellular Mismatch Repair Protein MSH2

**DOI:** 10.3390/v14050952

**Published:** 2022-05-02

**Authors:** Fei Ke, Renbao Wang, Zihao Wang, Qiya Zhang

**Affiliations:** 1 Institute of Hydrobiology, The Innovation Academy of Seed Design, Chinese Academy of Sciences, Wuhan 430072, China; wangrenbaovip@163.com (R.W.); wangzh@ihb.ac.cn (Z.W.); zhangqy@ihb.ac.cn (Q.Z.); 2 College of Modern Agriculture Sciences, University of Chinese Academy of Sciences, Beijing 100049, China

**Keywords:** iridovirus, ranavirus, MutS homolog 2, replication, transcription, knockout

## Abstract

As nucleocytoplasmic large DNA viruses, replication of ranaviruses (genus *Ranavirus*, family *Iridoviridae*) involves a series of viral and host proteins. We have described that the replication and transcription machinery of *Andrias davidianus* ranavirus (ADRV) which was isolated from the Chinese giant salamander contained host factors. Here, a new host factor, the MutS homolog 2 (MSH2), was proved as an important protein that participated in ADRV infection. Expression of *MSH2* was stable during ADRV infection in cultured cells and it localized at the cytoplasmic viral factories and colocalized with virus nascent DNA, indicating its possible role in virus genome replication. Investigation of the viral proteins that interacted with MSH2 by co-immunoprecipitation showed that *A. davidianus* MSH2 can interact with ADRV-35L (possible components associated with virus transcription), ADRV-47L (virus DNA polymerase), and ADRV-98R. Further knockdown *MSH2* expression by RNAi significantly reduced the late gene expression of ADRV. Additionally, *MSH2* knockout by CRISPR/Cas9 significantly reduced viral titers, genome replication, and late gene transcription of ADRV. Thus, the current study proved that ADRV can engage cellular MSH2 for its efficient genome replication and late gene transcription, which provided new information for understanding the roles of host factors in ranavirus replication and transcription.

## 1. Introduction

Ranaviruses are nucleocytoplasmic large DNA viruses that belong to the family *Iridoviridae* [1,2], which have been isolated from aquatic animals globally, such as bony fish, amphibians, and reptiles [3,4,5,6]. Because of the wide host range, ranaviruses represent a great threat to cultured and wild aquatic animals [7,8,9]. The sequenced ranaviruses possessed genomes with a unit size of 104–140 kbp, which can encode more than 100 proteins [1].

Ranavirus genome replication has been reported to occur in two-stages involving the nucleus and cytoplasm [10]. Although several viral proteins involved in ranavirus DNA replication have been predicted or investigated [11,12,13], the mechanism of ranavirus DNA replication still needs to be explored. *Andrias davidianus* ranavirus (ADRV) was isolated from diseased Chinese giant salamanders, which have a genome size of 106.7 kbp with 101 predicted open reading frames (ORFs) [6]. Because of the intracellular life cycle, virus replication involves not only viral proteins but also host proteins. In a recent study on the replication and transcription machinery of ranaviruses, we identified several viral and host proteins as potential proteins associated with viral nascent DNA by proteomic analysis of ADRV infected cells [14]. Among them, the host MutS homolog 2 (MSH2) was found but its function in virus infection was unknown.

MSH2 belonged to the DNA mismatch repair (MMR) proteins, which are highly conserved from lower to higher animals [15]. MSH2 along with the other MMR proteins such as MSH3 and MSH6 play important functions in maintaining the integrity of the genome in normal cells [16,17]. They can recognize the mismatches and insertion/deletion loops that occur in the genome and thus are needed for the repair of DNA replication errors [18]. The MMR proteins have been shown to be involved in the replication of the herpes viruses including herpes simplex virus 1 and Epstein-Barr virus [19,20], which are DNA viruses replicated in the nucleus. However, the function of DNA mismatch repair protein in ranavirus replication which mainly occurred in the cytoplasm is unknown.

In the present study, we explored the function of MSH2 in ADRV infection and replication by immunofluorescence, co-immunoprecipitation, RNA interference, and CRISPR/Cas9 knockout. The results showed that MSH2 can interact with virus proteins and localize in viral factories. Knockdown or knockout of MSH2 expression reduced virus genome replication and late gene expression.

## 2. Materials and Methods

### 2.1. Virus and Cells

ADRV and RGV (*Rana grylio* virus) that were maintained in our lab were used in the present study [6,21]. The Chinese giant salamander thymus cell (GSTC) line was the culture in the M199 medium supplemented with 10% fetal bovine serum at 25 °C until use [22]. Baby hamster kidney fibroblast cells (BHK-21) and human embryonic kidney (HEK293T) cells were grown in Dulbecco’s modified Eagle’s medium (DMEM) supplemented with 10% fetal bovine serum at 37 °C in 5% CO_2_ [14]. To prepare virus stocks, GSTC cells were infected with ADRV or RGV at an MOI of 0.1. Three days post-infection, the infected cells were collected, freeze-thawed, aliquoted, and kept at −80 °C until use.

### 2.2. MSH2 Gene Cloning and Plasmid Construction

Total RNA was extracted from GSTC cells with TRIzol Reagent (Thermo Fisher, Waltham, MA, USA) and the first strand of cDNA was synthesized with HiScript III 1st Strand cDNA Synthesis Kit (+gDNA wiper) (Vazyme, Nanjing, China) according to the manufacturer’s recommendations. The coding sequence of the *MSH2* gene was amplified with primers (5′-AGCCTGATGCTGATGT CTAGAATGGCGGTGCAACCC-3′/5′-ACCCTGAAGTTCTCAGGATCCTTATGCAGTAGTCTTTGTTCG-3′) by PCR based on the sequences from previous transcriptomic data [23] and then ligated into the pCGN-HAM vector by infusion cloning to generate 3HA-tagged MSH2 protein. The recombinant plasmids expressing 3Flag-tagged viral proteins were constructed as described previously [14]. The obtained plasmids were proved by DNA sequencing.

### 2.3. Western Blotting

BHK-21 cells were infected with ADRV at an MOI of 0.5. The cells were harvested at the indicated time points and subjected to Western blotting analysis as described previously [24]. A rabbit anti-MSH2 antibody (A8740, ABclonal, Wuhan, China) was used as the primary antibody, and the corresponding horseradish peroxidase (HRP)-conjugated goat anti-rabbit IgG (ABclonal, Wuhan, China) was used as the secondary antibody. Antibody binding was detected by chemiluminescence (Millipore, Burlington, MA, USA). Detection of β-actin was used as an internal control.

### 2.4. EdU Labeling and Immunofluorescence

EdU labeling was performed as described previously [14]. Briefly, BHK-21 cells plated on coverslips were infected with ADRV or RGV at an MOI of 0.5 and incubated at 28 °C. EdU (Invitrogen) was added to a final concentration of 10 μM at the times indicated. After continued incubation for 30 min, the cells were washed with phosphate-buffered saline (PBS), fixed with 4% paraformaldehyde, and then processed with a Click-iT Plus EdU Cell Proliferation Kit for Imaging (Thermo Scientific, Waltham, MA, USA) according to the manufacturer’s protocols. Alexa Fluor 488-azide was used in the kit to label the EdU. In the following immunofluorescence assay, the EdU labeled cells were then incubated with antibodies against MSH2 (rabbit anti-MSH2 antibody, ABclonal), followed by the Alexa Fluor 546 conjugated Donkey anti-Rabbit IgG (Invitrogen, Waltham, MA, USA). Cell nuclis were stained with DAPI. Images were collected on a Leica TCS SP8 confocal microscope.

### 2.5. Coimmunoprecipitation (co-IP) Assay

Co-IP assays were performed in HEK293T cells for their high transfection efficiency. The cells seeded in 6-well plates were cotransfected with 3Flag-tagged plasmid and 3HA-tagged plasmid (1.25 μg for each). An empty vector was used as a control. The cells were collected at 36 h post-transfection (hpt) and lysed with radioimmunoprecipitation assay (RIPA) buffer (Beyotime, Shanghai, China) containing PMSF and protease inhibitor (MCE, Shanghai, China). The cell lysates were centrifuged and the supernatants were incubated with anti-Flag affinity gel (Sigma) for 4 h at 4 °C. The precipitates were washed with ice-cold PBST five times and subjected to Western blot as described above. Commercial anti-Flag (Sigma, Waltham, MA, USA) and anti-HA (CST, Danvers, MA, USA) antibodies were used.

### 2.6. RNA Interference

Three siRNAs targeting *Mesocricetus auratus MSH2* (GenBank accession number: XM_005077304) were designed and synthesized (GenePharma, Shanghai, China), which were transfected into BHK-21 cells at a concentration of 25 nM using Lipofectamine RNAiMAX (Thermo Fisher, Waltham, MA, USA). A commercial synthesized NC siRNA was used as a negative control. The transfected cells were collected at 36 hpt to perform a Western blot analysis. Alternatively, the cells were infected with ADRV at an MOI of 0.5 and collected at the indicated time points to perform RNA or DNA extraction. The equences of the siRNAs are listed in Table 1.

### 2.7. MSH2 Knockout Cells Construction and Virus Infection

*MSH2* knockout cells were established by the CRISPR-Cas9 method. Sequences targeting the *Mesocricetus auratus MSH2* gene were designed and ligated into the plasmid pU6gRNA1Cas9EGFPU6gRNA2 (GenePharma, Shanghai, China) which contained two U6 promoters and can express two gRNAs, Cas9, and EGFP simultaneously. The two targeted sequences in the *MSH2* gene were as follows: 5′-agaagtcgccgcggtcgaag-3′ (108–127, antisense) and 5′-cttctacacggcgcacggcg-3′ (123–142, sense). The two gRNAs would target the sequences from 108 to 142 bp of the *MSH2* gene, which could enhance the editing efficiency and accuracy of one gRNA. The obtained plasmid was transfected into BHK-21 cells. At 48 hpt, the cells expressing EGFP were sorted into 96-well plates by flow cytometer and each well only contained one cell. The plates were cultured at 37 °C until clones formed. Individual clones were further transferred into 6-well plates and then identified by DNA sequencing and Western blotting. The finally obtained cell clone was named BHK-ΔMSH2.

For virus infection, wild-type BHK-21 or the BHK-ΔMSH2 were seeded into 24-well plates in the same cell numbers for each well. Then, the cells were inoculated with ADRV or ADRV_46R-3Flag_ [14] at an MOI of 0.5. After incubation for 1 h, the medium containing the virus was moved and the fresh medium was added and incubated at 28 °C. At indicated time points, the cells were observed under a fluorescence microscope or collected for viral titer assay, or RNA and DNA extraction respectively.

### 2.8. Virus Titer Assay

Virus titers of the samples collected above were determined on triplicate monolayers of GSTC cells by using the 50% tissue culture infectious dose (TCID_50_) assays as described previously [25].

### 2.9. Real-Time RT-PCR for Virus Gene Expression

RNA was extracted from the collected cells with TRIzol Reagent (Thermo Fisher, Waltham, MA, USA). First-strand cDNA synthesis was performed using HiScript III RT SuperMix for qPCR (+gDNA wiper) (Vazyme, Nanjing, China) as per the manufacturer’s recommendations. RT-qPCR was conducted using a CFX96 Touch Real-Time PCR Detection System (BioRad, Hercules, CA, USA). Each RT-qPCR mixture contained 10 μL of SYBR Premix (2×), 0.5 μL of forward and reverse primers (for each primer), 1 μL of cDNA, and 8 μL of ultrapure water. The β-actin gene was used as an internal control. The RT-qPCR conditions were as follows: 95 °C for 10 min; 40 cycles of 95 °C for 15 s, 60 °C for 1 min; and a melt curve analysis at 95 °C for 15 s, 60 °C for 1 min, and 95 °C for 15 s. The mRNA relative expression ratios of the treated group versus that of the control group were calculated by the 2^−ΔΔCT^ method. The primers used in the previous study were used [14].

### 2.10. Real-Time PCR for Virus Genomic Copies

DNA was extracted with the Takara MiniBEST Universal Genomic DNA Extraction Kit (TakaRa, Maebashi, Japan). Virus genomic copies were determined by detecting the viral major capsid protein gene (*MCP*) copy numbers with quantitative real-time PCR (qPCR), which was conducted in a CFX96 Touch Real-Time PCR Detection System (BioRad) as described above. The plasmid pMD18T-MCP used previously was used as a template to construct the standard curve [14]. The MCP amount (genome copy number) was calculated with the standard curve.

### 2.11. Statistical Analysis

All data represent results of three biological replicates and were analyzed with Student’s t-test in GraphPad Prism 9 or Microsoft Excel. Significant differences are marked with a (0.01 < *p* < 0.05) or b (*p* < 0.01).

## 3. Results

### 3.1. MSH2 Was Expressed during ADRV Infection and Localized with Viral Nascent DNA

**Figure 1:**
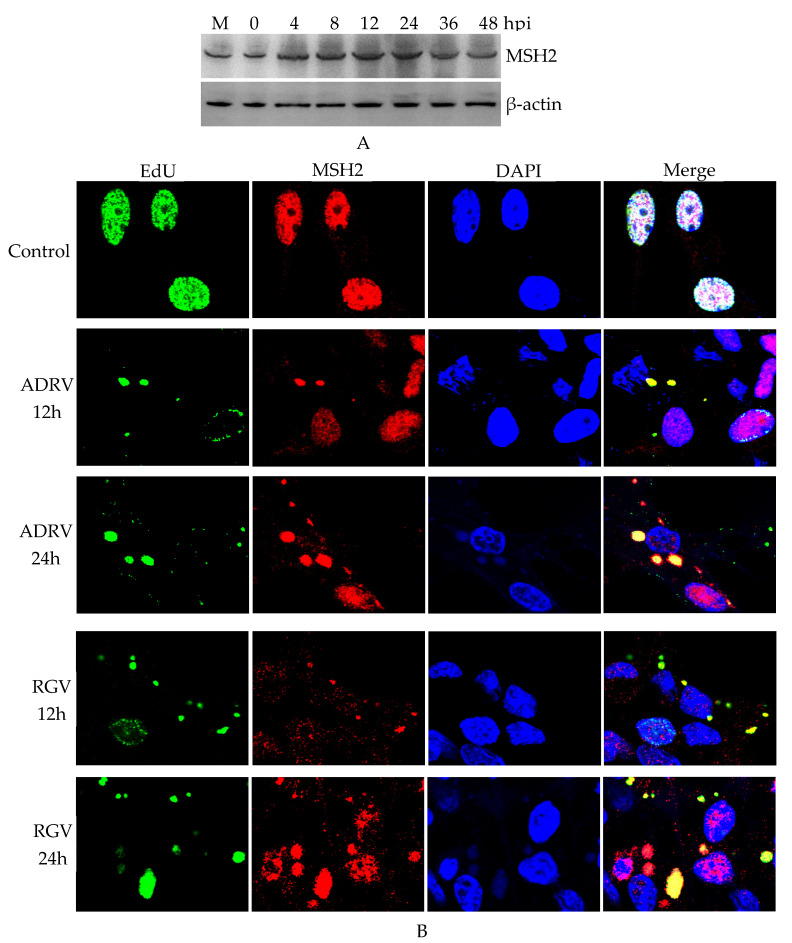
Expression and subcellular localization of MutS homolog 2 (MSH2) in virus-infected BHK-21 cells. (**A**). Western blot analysis of MSH2 expression in *Andrias davidianus* ranavirus (ADRV) infected cells. (**B**). Immunofluorescence detection of MSH2 in ADRV and *Rana grylio* virus (RGV) infected cells. Nascent DNA was stained by EdU (green color). MSH2 was stained by rabbit anti-MSH2 antibody (Red color). DAPI (blue color) was used to stain the nuclei and viral factories. Objective magnification ×63.

The expression of MSH2 during ADRV infection of BHK-21 cells was examined by Western blot analysis. The results showed that bands corresponding to MSH2 were stably detected among the examined times (0–48 hpi) (Figure 1A), which indicated that the expression of MSH2 was not inhibited by ADRV infection.

Subcellular localization of MSH2 was further examined by immunofluorescence. EdU was used to label the virus nascent DNA and viral factories. As shown in Figure 1B, in the cells without infection (control), EdU (green color) was detected in the nucleus, indicating a replicating cell. MSH2 was also localized in the nucleus in the normal cells. However, in ADRV infected cells, most of EdU labeled loci were localized in the cytoplasm in an aggregated form, indicating the replicating viral DNA. MSH2 also appeared in the cytoplasm and colocalized with the cytoplasmic EdU and DAPI stained viral factories in the infected cells. Most of the fluorescence signal and size of the cytoplasmic colocalized EdU and MSH2 increased from 12 to 24 hpi. We also examined the subcellular localization of MSH2 in BHK-21 cells infected by another ranavirus RGV, which showed similar results as the ADRV infected cells (Figure 1B). Collectively, expression and colocalization assays indicated that host MSH2 could participate in ADRV infection.

### 3.2. MSH2 Interacted with Viral Proteins

The MSH2 localized in the cytoplasmic viral factories in infected cells while its normal localization was the nucleus, which indicated possible interactions between MSH2 and viral proteins. We further tried to explore the interacted viral proteins. With previous transcriptome data, the MSH2 coding sequence was first cloned from GSTC cells. The ORF of *MSH2* of GSTC (gMSH2) has a size of 2796 bp (GenBank accession number ON086763), encoding a protein with 931 aa.

**Figure 2:**
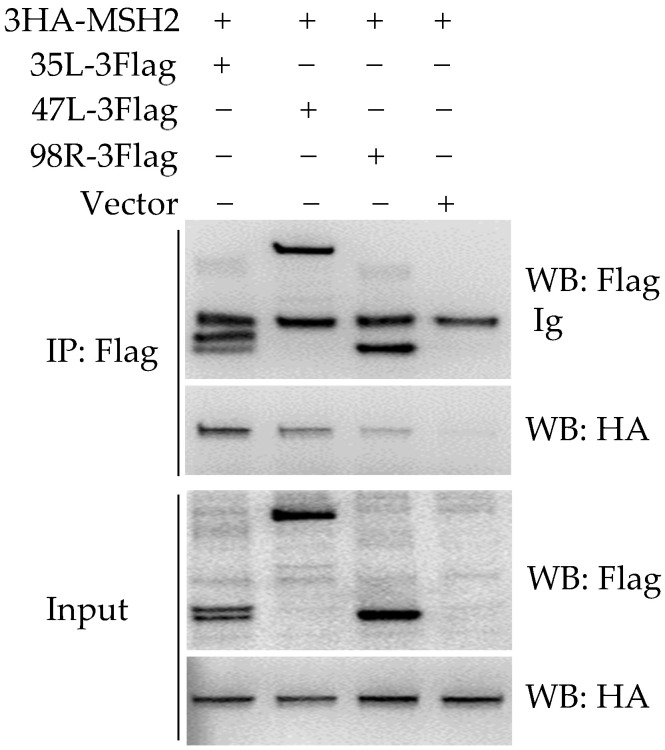
Detection of the interaction between MSH2 and ADRV-35L, ADRV-47L, or ADRV-98R. Cell lysates (Input) from HEK293T cells cotransfected with plasmids expressing the indicated proteins and immunoprecipitated (IP) protein complexes were subjected to Western blot analysis with anti-Flag or anti-HA antibody.

Using the NanoLuciferase complementation assay established previously [14], a relatively higher luciferase was found when cotransfection of gMSH2 with ADRV 35L, 47L, or 98R (data not shown), indicating possible interactions among them. Co-IP was further performed to verify the interactions. As shown in Figure 2, the positive bands corresponding to 3HA-MSH2 were detected in immunoprecipitated (IP) complex (affinity by anti-Flag gel) from the samples co-transfected with 35L-3Flag, 47L-3Flag, and 98R-3Flag respectively. In the three combinations, the IP band of 3HA-MSH2 was strongest in 35L-3Flag cotransfected cells and weakest in 98R cotransfected cells.

### 3.3. Knockdown of MSH2 Affected Viral Late Gene Expression

**Figure 3:**
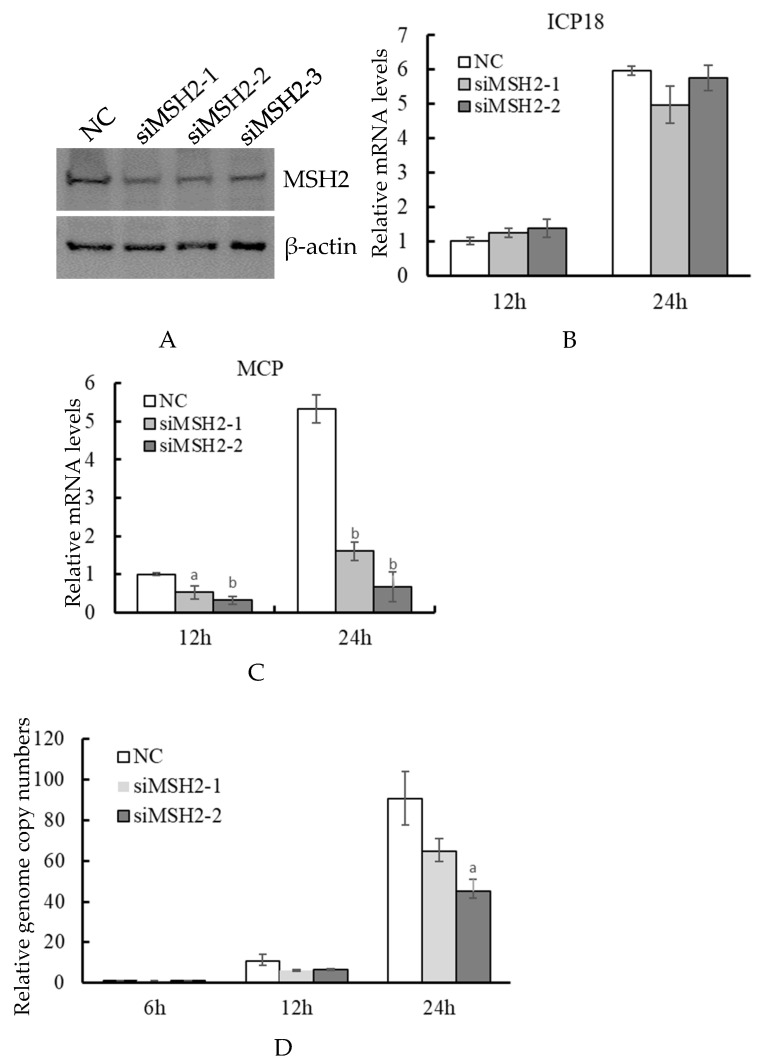
Determination of the effect of MSH2 knockdown on ADRV infection. siRNAs targeting MSH2 were transfected into BHK-21 cells. The transfected cells were subjected to Western blotting analysis (**A**) or infected with ADRV. Expression of ADRV immediate early gene *ICP18* (**B**) and late gene *MCP* (**C**) were determined by RT-qPCR. The virus genome copy numbers were determined by quantitative real-time PCR with the standard curve. The virus genome copy number in NC transfected cells at 6 hpi was set as 1 in the figure (**D**). Significant differences (versus NC at the time point) are marked with a (0.01 < *p* < 0.05) or b (*p* < 0.01).

We further performed an RNAi assay to test the effect of MSH2 knockdown on virus infection. In the three siRNAs targeted to MSH2, siMSH2-1 and siMSH2-2 showed a relatively higher interference efficiency than siMSH2-3 as revealed by Western blot analysis (Figure 3A). siMSH2-1 and siMSH2-2 were used in the following analysis. RT-qPCR showed that there was no significant difference in the expression of viral immediate-early gene *ICP18* between the two siRNAs and control NC at 12 and 24 hpi (Figure 3B). However, the two siRNAs both significantly (*p* = 0.010401 and 0.000128 for siMSH2-1, *p* = 0.000414 and 0.000109 for siMSH2-2, at 12 and 24 hpi respectively) reduced the expression of the viral late gene *MCP* (Figure 3C). The viral genome copy numbers were further detected by quantitative qPCR. The results showed that there was no significant difference between the siRNAs and NC at 6 hpi, and the viral genome copy numbers in siRNA transfected cells were less than that of NC at 12 and 24 hpi, but the significant difference was only observed in siRNA-2 transfected cells at 24 hpi (*p* = 0.017361) (Figure 3D).

### 3.4. Construction of MSH2 Knockout Cells

**Figure 4:**
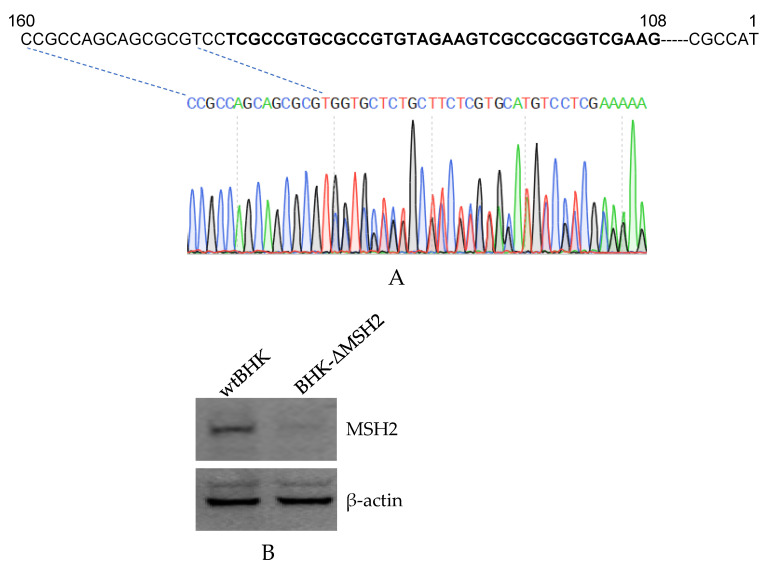
Establishment of *MSH2* knockout BHK cells by CRISPR/Cas9. (**A**). DNA sequencing confirmation of the mutated regions in the *MSH2* gene. The sequences with bold font are the targeted sequences by the two gRNAs. The PCR products were sequenced from the reverse direction and the sequence chromatogram showed the sequence from position 160 bp of the gene. (**B**). Western blot analysis of MSH2 expression in wild-type BHK-21 (wtBHK) and mutated BHK (BHK-ΔMSH2) cells. Detection of β-actin was used as an internal control.

Considering the efficiency of the MSH2 RNAi was not high enough, there were still a large amount of expressed MSH2, which could affect the RNAi results, therefore, we tried to establish *MSH2* knockout cells by CRISPR/Cas9. A BHK-21 cell lacking the expression of *MSH2* (BHK-ΔMSH2) was finally obtained. The sgRNAs targeted sequence was located from 108 to 142 nt in the *MSH2* mRNA. DNA sequencing showed that the N terminal region of the *MSH2* coding sequence was mutated (Figure 4A). The MSH2 corresponding band was not detected in the BHK-ΔMSH2 cells (Figure 4B).

### 3.5. MSH2 Knockout Inhibited ADRV Infection

**Figure 5:**
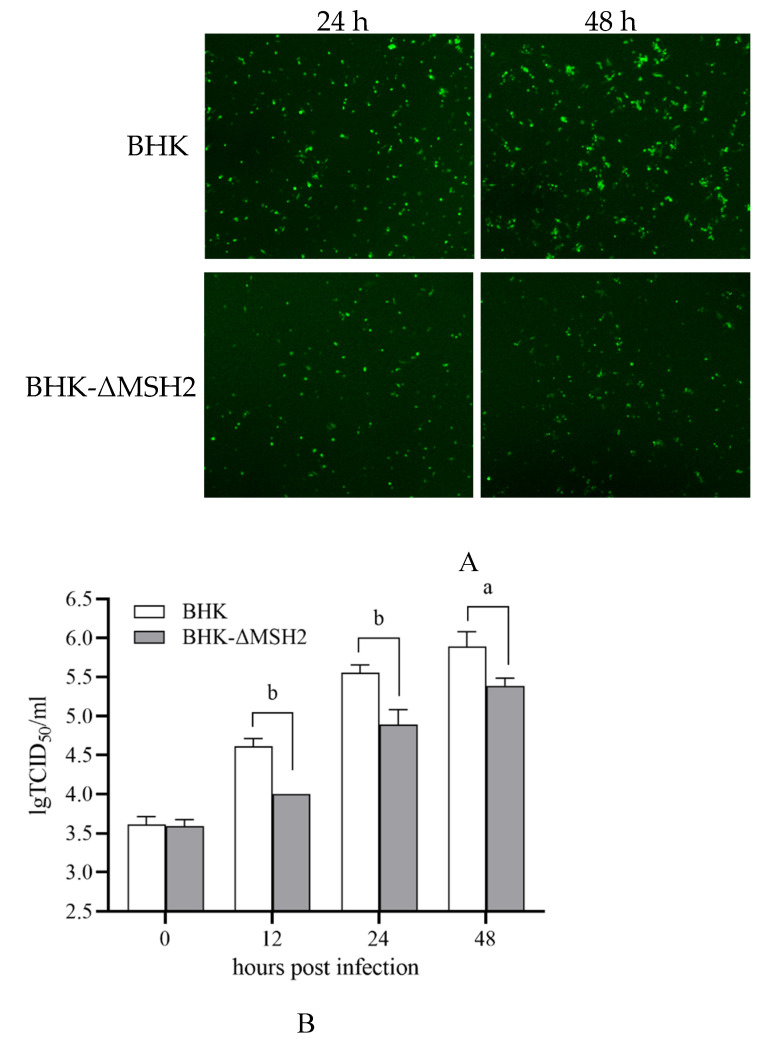
*MSH2* knockout inhibited ADRV infection. (**A**). Fluorescence observation of the wild type BHK and BHK-ΔMSH2 cells infected with ADRV expression EGFP at 24 and 48 hpi. Objective magnification ×4. (**B**). Determination of virus titers in the two cells infected with ADRV by TCID_50_ methods showed that ADRV titers were reduced in *MSH2* knockout cells. Significant differences are marked with a (0.01 < *p* < 0.05) or b (*p* < 0.01).

ADRV infection in the BHK-ΔMSH2 cells was examined by infecting the cells with a recombinant ADRV expression EGFP. The results showed that EGFP expression was reduced in BHK-ΔMSH2 cells compared to wild-type BHK cells at the examined time points (24 and 48 hpi) (Figure 5A). The virus titers at different times (0, 12, 24, 48 hpi) were further determined by the TCID_50_ assay. The virus titers between wild-type BHK and BHK-ΔMSH2 at 0 hpi were nearly equivalent, but the virus titers from BHK-ΔMSH2 were significantly less than that from wild-type BHK at 12 (*p* = 0.000413), 24 (*p* = 0.005739), and 48 (*p* = 0.015251) hpi (Figure 5B), which indicated that *MSH2* knockout inhibited ADRV infection.

### 3.6. MSH2 Knockout Reduced Viral Genome Replication and Late Gene Expression

**Figure 6:**
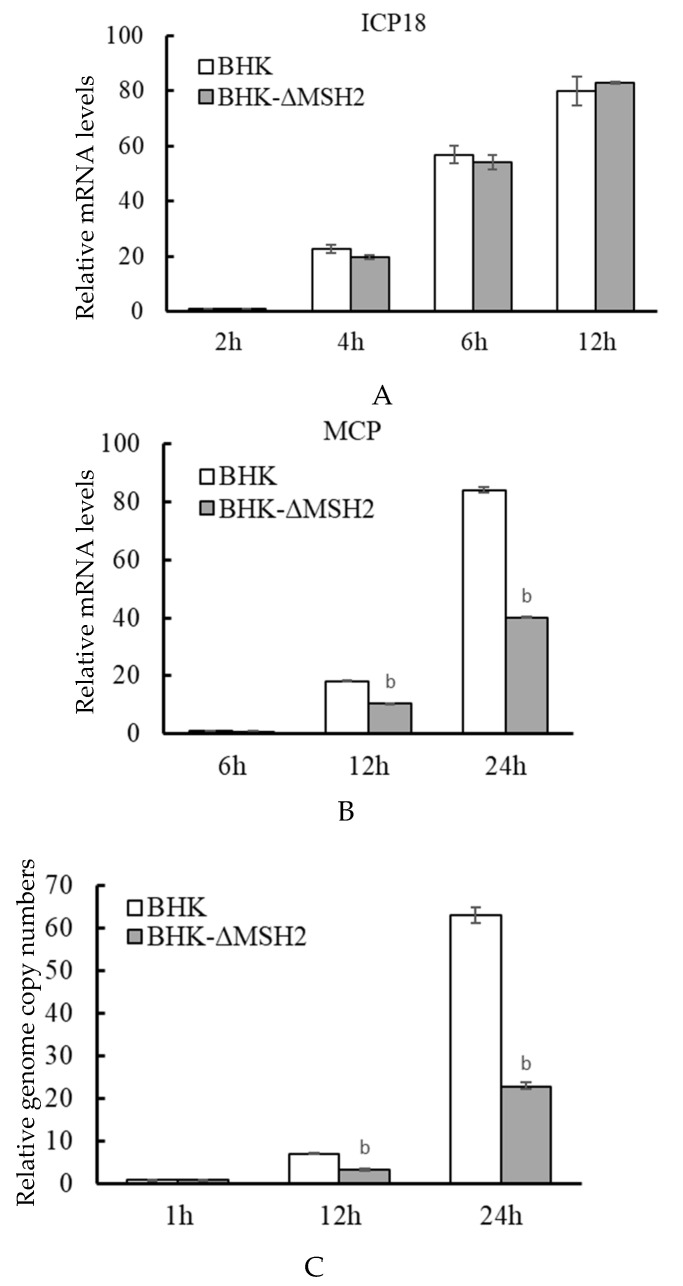
*MSH2* knockout inhibited genome replication and late gene expression of ADRV. Expression of the immediate-early gene *ICP18* (**A**) and late gene *MCP* (**B**), and the virus genome copy numbers (**C**) were determined as described above. Significant differences (ΔMSH2 versus BHK-1 at the time point) are marked with b (*p* < 0.01).

The virus gene expression and genome replication were further examined in the BHK-ΔMSH2 cells. Similar to that observed in RNAi assays, the expression of ADRV immediate early gene *ICP18* was not affected in BHK-ΔMSH2 cells compared to wild-type BHK cells at the tested time points (2, 4, 6, and 12 hpi) (Figure 6A), and the expression of the late gene *MCP* was significantly reduced at 12 (*p* = 0.008371) and 24 hpi (*p* = 0.000757) in the BHK-ΔMSH2 cells (Figure 6B). 

The virus genome copy numbers were determined simultaneously. No significant difference was found between the BHK-ΔMSH2 and wild-type BHK cells at 1 hpi, which indicated that *MSH2* knockout had no effect on virus attachment and entry. However, the viral genome copy numbers from BHK-ΔMSH2 cells were significantly reduced at 12 (*p* = 0.00397) and 24 hpi (*p* = 0.000271) compared to the wild type BHK cells (Figure 6C). These results indicated that *MSH2* knockout affected ADRV genome replication and late gene expression.

## 4. Discussion

Host DNA repair protein MSH2 was detected in our previous study on ranavirus replisome and transcription complex [14], although genome sequence analysis has revealed that iridoviruses including ranaviruses encoded protein potentially involved in DNA repair, such as the RAD2 homolog [6,26,27,28,29,30,31]. In the present study, the host MSH2 was cloned and characterized as an important factor for ADRV replication, which was thefirst to report the host mismatch repair protein involved in iridovirus infection. 

Subcellular localization revealed that MSH2 localized in cytoplasmic viral factories is the place where viral DNA replication occurred [32]. Most of the viral nascent DNA colocalized with MSH2, which indicated the association between MSH2 and virus DNA replication. However, sometimes, there were still some punctate areas emitting weak green color (nascent DNA) and a strong red color (MSH2), which hinted that the nascent DNA synthesis and MSH2 localization might be asynchronous steps. 

MSH2 was further shown to have interactions with ADRV proteins 35L, 47L, and 98R. The function of ADRV-98R was unknown up to now. In our previous study, ADRV-35L was found to be associated with the virus transcription complex [14], and ADRV-47L is the virus DNA polymerase [6,14]. The interactions hinted that MSH2 might participate in virus genome replication and gene transcription, but the exact mechanism needs further research.

RNAi assay revealed that knockdown of MSH2 inhibited the expression of the viral late gene but not the immediate-early gene, which indicated that MSH2 participated in a later step in the virus life cycle. It has been reported that silencing of MSH2 inhibited early gene expression in HSV infected cells [20], which is a virus replicating in the cell nucleus. The effect on virus DNA replication was not obvious in the present RNAi assay, which prompted us to deplete the MSH2 expression, and finally, the BHK-21 cells that lacked MSH2 expression were obtained. Deletion of MSH2 expression significantly affected the virus’ late gene expression and DNA replication. However, it has been reported that full late gene expression requires prior DNA synthesis (nascent DNA) [1]. So, the transcription of late genes of ranaviruses started after virus DNA replication [33], and inhibition of virus DNA replication would affect the transcription of late genes. Thus, it is not clear whether MSH2 participated in virus late gene transcription directly.

In addition, BHK-21 cells have been used in the research of ranavirus infection, such as frog virus 3 [34,35]. Our previous experiments have shown that ADRV and RGV infected efficiently in BHK-21 cells. The cells have been used in the investigation of the transcription complex of ADRV [14]. So, we performed the subcellular localization, knockdown, and knockout assays of MSH2 in BHK cells for the availability of commercial antibodies and the feasibility of gene knockout by CRISPR/Cas9, although the MSH2 was initially found in ADRV infected GSTC cells as the viral nascent DNA associated protein. The results from BHK-21 cells also suggested the importance of MSH2 in ADRV replication in different cells.

Therefore, the present study confirmed that cellular MSH2 was hijacked by ADRV to facilitate its efficient replication and transcription by using subcellular localization, co-IP, and gene knockdown/knockout assays, which could occur in other ranavirus infection for the subcellular co-localization was also observed in RGV infection. Thus, the study provided new insights into the understanding of ranavirus replication and ranavirus-host interactions.

## Figures and Tables

**Table 1:** siRNA sequences used in the present study.

siRNA	Sequences (5′-3′)	Positions in *MSH2* Gene
siMSH2-1	GGUUCGUCAGUAUAGAGUUTT (sense)	282–300 bp
	AACUCUAUACUGACGAACCTT (antisense)	
siMSH2-2	GCUUUGCUCACGUCUCAAATT (sense)	1820–1838 bp
	UUUGAGACGUGAGCAAAGCTT (antisense)	
siMSH2-3	GCACUAACUAGUGAAGAAATT (sense)	2410–2428 bp
	UUUCUUCACUAGUUAGUGCTT (antisense)	
NC	UUCUCCGAACGUGUCACGUTT (sense)	
	ACGUGACACGUUCGGAGAATT (antisense)	

## Data Availability

The *MSH2* sequence of GSTC cells obtained in the study has been submitted into GenBank under accession number ON086763.
